# Effects of sibling competition and physiological and behavioural differences on problem-solving of wild cavies (*Cavia aperea*)

**DOI:** 10.1007/s10071-026-02094-w

**Published:** 2026-08-11

**Authors:** Sabine Kraus, Anja Guenther

**Affiliations:** 1 Department of Animal Behaviour, Bielefeld University, Bielefeld, Germany; 2 Department of Behavioural Ecology, Bielefeld University, Bielefeld, Germany; 3 Zoology and Animal Ecology, Institute of Biology and Chemistry, Hildesheim University, Hildesheim, Germany

**Keywords:** Bad-competitor hypothesis, Size rank, Personality, Early development, Cortisol, Metabolism

## Abstract

Behavioural innovations can be crucial for animals to adjust to changing environments and to exploit novel resources. While ecological and evolutionary implications of innovativeness have been investigated intensely, the proximate mechanisms underlying this behaviour are less well understood. Intrinsic individual differences arising from differences in competitive abilities have been hypothesised to drive differences in innovation propensity (bad-competitor hypothesis). We here investigated the relationship between early-life competition among siblings as well as effects of several physiological and personality traits on innovation propensity in young wild cavies (*Cavia aperea*). Our study aimed to test whether competitive situations in early life, such as being a small sibling or being born in larger litters, enhanced innovation propensity. Furthermore, we tested if intrinsic physiological or behavioural characteristics differed between solvers and non-solvers. We found that neither size rank nor litter size influenced innovation propensity in young cavies at independence. Likewise, physiological traits such as cortisol level, heart rate, and resting metabolic rate did not differ systematically between solvers and non-solvers, although heart rate and resting metabolic rate were repeatable. However, individuals that interacted more with novel objects and innovation tasks were more likely to solve them. Our results suggest that innovation propensity is not shaped by early-life competition or physiological differences but may rather develop consistent individual differences later in life, potentially by competition for mating opportunities.

**Supplementary Information:**

The online version contains supplementary material available at 10.1007/s10071-026-02094-w.

## Significance statement

Behavioural innovation, often measured by problem solving tests, often helps animals to cope with environmental change and adjust to novel environments. How and when individual differences in innovative propensities arise and are shaped during development remains currently unknown. Poor competitive abilities are generally suggested to drive enhanced innovation propensity. Here, we tested if sibling competition during early life or physiological and behavioural differences drive innovativeness in the wild cavy (*Cavia aperea*). Innovation propensity was not shaped by early-life competition or physiological traits. Individuals interacting more with novel objects and innovation tasks were more likely to innovate. Our study contributes to a more comprehensive understanding of the role of behavioural and physiological traits of animals for the propensity to innovate.

**Supplementary Information:**

The online version contains supplementary material available at 10.1007/s10071-026-02094-w.

## Introduction

Behavioural innovations have recently been recognised as an important characteristic that allows animals to adjust to human-induced fast environmental change (Sol et al. 2002), to mitigate colonisation success in novel habitats and to overcome challenging environmental conditions (Kozlovsky et al. 2015). Bullfinches (*Loxigilla barbadensis*) for example have developed a novel technique to open sugar packets from restaurant terraces (Ducatez et al. 2013; Reader et al. 2002), which enables them to exploit an important resource opportunity inaccessible to most other animals. While the implications of innovativeness are increasingly appreciated among evolutionary ecologists, the proximate mechanisms, i.e., ontogenetic and/or physiological mechanisms sensu Tinbergen (1963) responsible for the innovativeness of an individual are less well understood.

Within species, individuals often vary in their propensity to innovate – or problem-solve, a proxy which is often used in experimental designs to measure innovation (Morand-Ferron et al. 2011; Griffin and Guez 2014; Rowell et al. 2021). Variation between individuals could arise due to intrinsic (e.g., genetic, neuroendocrine or aging) and/or extrinsic (e.g. environmental) factors (Rowell et al. 2021). Socio-ecological environmental conditions experienced during development, especially during early life, may profoundly influence whether an individual develops capacity in innovation. Social effects during early life stages can be positive (e.g. by receiving high levels of maternal care) or negative (e.g. by sibling competition), especially in species with extended parental care. Suboptimal parental care, leading to offspring in poorer condition should, according to the necessity drives innovation hypothesis and the bad competitor hypothesis, promote innovative problem-solving proficiency (Reader and Laland 2003; Amici et al. 2019). So far, only one study investigated how the early social life affects problem-solving later in life. This study on fawn-footed mosaic-tailed rats (*Melomys cervinipes*) suggested that the level of maternal care and therefore offspring condition, did not influence later innovation propensity (Rowell and Rymer 2022). Among siblings, competition for limited resources such as maternal care or milk (mammalian offspring), is an important developmental mechanism shaping individual differences in behaviour, physiology and even fitness (Dimitsantos et al. 2007; Rödel et al. 2009; Rödel and Meyer 2011). Individuals that are relatively large (compared to their siblings) typically have a competition advantage (Stockley and Parker 2002). If and how such sibling effects influence innovative problem-solving long-lasting has not yet been investigated.

Individually consistent traits, i.e. personality traits (Réale and Dingemanse 2012) that are often correlated with innovative problem-solving (Griffin and Guez 2014), such as boldness or exploration tendencies, are on the other hand, well-known to be shaped by various early-life experiences (Stamps and Groothuis 2010; Groothuis and Trillmich 2011). For example, rats and other rodents that received less maternal care (often specifically less comforting behaviours such as licking and grooming by the mother), behave less bold and explorative and show stronger responses to stress (Champagne and Meaney 2007; Beery and Francis 2011). The quantity and quality of parental care has been demonstrated to alter the neural circuits of the limbic system (hypothalamus, hippocampus, amygdala), which constitute the functional hub underlying the formation of emotions (Callaghan and Tottenham 2016). Multiple neuro-endocrine pathways are programmed by maternal care (Meaney 2001; Champagne 2008), thereby influencing behavioural development of stress-coping and anxiety-like behaviour as well (Cameron et al. 2005). Direct (e.g. by aggressive interactions) or indirect (by scramble) competition among littermates is known also to shape behaviour and physiology long-lasting (Hudson et al. 2011). Competition for maternal milk for example facilitates aggressive attacks among piglets (*Sus scrofa*) using their already well-developed sharp incisors, especially in large litters, where typically not all piglets survive (Andersen et al. 2011). In the domesticated guinea pig (*Cavia porcellus*), overt aggression is only seen in rare cases but pup scramble for mothers’ teats, usually with larger pups gaining the advantage (Fey and Trillmich 2008). In cats (*Felis sylvestris*) and meerkats (*Suricata suricata*), littermates actively compete over food around weaning, often with aggressive interactions (Hodge et al. 2007; Gonzalez et al. 2018). Rabbit pups compete, mostly via scramble competition, for the central positions in the litter huddle because those are thermoregulatory advantageous (Bautista et al. 2013), resulting in better body condition (e.g. García-Torres et al. 2015) and long-term stable behavioural differences (Reyes-Meza 2011). In laboratory rats, it has been shown that relatively high within-litter body-mass was associated with lower levels of stress-hormones (Rödel et al. 2010) and smaller siblings were less bold at weaning compared to their litter mates (Rödel and Meyer 2011).

Here, we tested if young individuals directly after independence consistently differ in their propensity to innovate. We used a medium-sized, precocial rodent, *Cavia aperea*, as test subjects. These animals have small litters of one to four pups and pups are able to locomote and feed on solid food within a few minutes after birth (Rood and Weir 1970). Pups are weaned after about three weeks and are then independent of their mother. Reproduction may in rare cases start as early as three months of age but usually only starts later when animals have grown considerably (Kraus et al. 2024). The precocial animals differ in size/body mass directly after birth due to differential allocation of maternal resources during pregnancy (Schumann et al. 2014). During pregnancy, heavier pups are located closer to the cervix and receive more nutrients through the maternal blood flow (Schumann et al. 2014).

Cavy mothers have only two teats, therefore, offspring compete for access to milk via scramble competition (Fey and Trillmich 2008). As a result, smaller siblings are behaviourally less proactive and explorative (measured in forced Open Field, voluntary exploration and novel object tasks: Guenther and Trillmich 2015; Kraus et al. 2020). Initial size differences among siblings (i.e., size rank within litter effects) persist across the lifetime of individuals and affect onset of maturation as well as reproductive success (Kraus et al. 2024). Similarly, cavy pups also differ in various physiological aspects, e.g., the smallest pups in a litter had highest stress hormone concentrations, while others as for example resting metabolic rates, did not differ between pups of different size ranks (Guenther and Trillmich 2015). In addition, competition is stronger in larger litters (Rehling and Trillmich et al. 2008). In accordance with the bad-competitor hypothesis, we hypothesised that small siblings within litters, which suffer most from early sibling competition, would be better at behavioural innovations than their larger siblings. Furthermore, we expected individuals born in larger litters to show innovation more likely than individuals born into small litters.

Independently of or in interaction with such early life effects on competitive ability, intrinsic behavioural tendencies, i.e., personality traits, have been suggested to mediate behavioural innovation. Novelty for example, elicits two opposite tendencies: aversion (neophobia) but also attraction (neophilia) (Greenberg 2003). Neophilia facilitates information gathering while neophobia protects the animal from potential dangers (Greenberg 2003; Griffin and Guez 2014). Across taxa, individuals vary consistently in their responses to novel places and objects (Brown et al. 2013; Mazza et al. 2021).

Reaction to novel objects or places and neophobia/neophilia affect how individuals encounter, approach and interact with novel aspects in their environment. Thus, these traits may affect the likelihood to encounter and engage with novel situations and, consequently, the likelihood to innovate if the opportunity arises (Reader and Laland 2003). Bolder individuals (often measured as an approach towards an unknown object) have been reported to be better innovators (Greenberg 2003; Griffin and Guez 2014; Amici et al. 2019), although evidence is mostly limited to bird species. In rodents, evidence is mixed so far. While there was no direct link between innovation and boldness (measured as distance covered in an open field test) in house mice (*Mus musculus*), bolder mice were more likely to encounter innovation possibilities (Vezyrakis et al. 2025). In striped field mice (*Apodemus agrarius*), bolder animals (measured as latency to enter an unknown area) were better in solving novel problems and innovate (Mazza and Guenther 2021). We thus tested if solvers and non-solvers differed in boldness when confronted with novel objects, novel arenas or when being handled. Given the mixed evidence from other rodent species, we did not make clear predictions about the relationship.

Last, physiological responses could also contribute to individual differences in innovation (Rowell et al. 2021, 2022). Two opposing hypotheses predict effects of the physiological state on innovation propensity. On the one hand, poor physiological conditions, often of animals with low competitive abilities, may promote innovations (Reader and Laland 2003). On the other hand, superior physiological condition may ensure greater cognitive capacity and thereby better problem-solving and learning performance (Bókony et al. 2014). House sparrows (*Passer domesticus*) with lower corticosterone concentrations for example, solved complex problems faster than birds with higher corticosterone concentrations (Bókony et al. 2014). In contrast, horses (*Equus callabus*) with higher corticosterone levels were better at innovating (Esch et al. 2019). A similar reasoning might apply to the increase of heart rates in response to a mild stressor, a physiological parameter that provides an instant proxy for the activation of the autonomous nervous system (Spodick et al. 1992). Such states of arousal have been linked to behavioural and cognitive performances in various species (Wascher 2021), hence, we might expect solvers and non-solvers to differ in heart rate response to a mild stressor (here handling). We here tested if solvers differ from non-solvers in their cortisol concentrations as well as heart rate responses to handling stress. Contradictory evidence from the literature does not permit a clear prediction of the relationship.

Adrenocortical activity is also tightly linked to energetic states via the energy homeostasis and energy metabolism (de Guia et al. 2014). To our knowledge, no study has yet tested directly whether metabolic rates differ between solvers and non-solvers. We might however, expect similar differences as predicted for glucocorticoid hormones, i.e., animals with a high metabolic rate might be more likely to innovate to fulfil their energetic requirements. Opposingly, animals with low metabolic rates might be able to spare the energy and time to invest into innovation, i.e. excess of energy (Amici et al. 2019). As a third physiological parameter we therefore tested if resting metabolic rates differed between solvers and non-solvers.

## Methods

### Animals and housing

All animals were derived from an outbred captive colony of wild cavies (*Cavia aperea*).

For breeding, adult females were relocated individually from outside enclosures, where they were kept when not involved in experiments, to 1 m² indoor enclosures in our facility one week before the onset of breeding to allow them to adjust to the change in housing conditions. Inside enclosures were equipped with wood chips, a shelter, a rough stone, a feeding dispenser and a water bottle. Always two females that were familiar with each other were housed next to each other, separated by a grid that allowed olfactory, visual and acoustical communication. This grid was exchanged by a solid wall when males were introduced for breeding because adult males react highly aggressive towards each other. Males were left with females for four weeks (about two oestrus periods) to ensure successful mating. Pregnancy of the precocial cavy lasts 59–62 days and males do not show parental care. After males were removed, the solid wall was once again exchanged by a grid wall to allow females social contact. In total, 32 breeding pairs were set-up.

Starting 58 days after the introduction of males, we checked enclosures daily for newborn pups, 29 mothers delivered in total 69 pups. When pups were found, we gently caught the juveniles, weighed them and gave them fur cuts for individual recognition. Pups remained with their mother for 21–22 days of age, when we weaned them, marked them permanently with a subcutaneous pit tag (ID 100, TROVAN, passive transponder system, Euro ID, Weilerswist, Germany) and assigned them to same-sex pairs of unrelated animals. At this time, we took another body mass measurement which we used to calculate the growth rate (mass weaning – mass birth/ 21 or 22 days).

All animals received hay and guinea pig chow (Höveler, Germany) as well as water *ad libitum*. Once a week, water was supplemented with Vitamin C (1 g/l) and three times a week, the animals received fresh food such as carrots, bell pepper or salad. Room temperature was maintained at 20 ± 2 °C (within the thermoneutral zone), while outside temperature varied naturally throughout the year. Inside, natural day light (through three large windows) was supplemented with artificial light (12 L:12D) throughout the experiment.

### Experimental setup

All animals underwent 8 different experimental procedures twice. The first round of personality tests and measurement of physiological variables was conducted directly after weaning, at an age of 23–24 days and was finished within four weeks. During these four weeks, we measured the response to an unknown object in a Novel object test, the response to an unknown environment in an Open Field test and the response to handling in a Struggle test. In addition, we measured the heart rate, took a blood sample to determine baseline plasma cortisol concentrations and measured resting metabolic rate. After the completion of the first test round, the animals were given two weeks break. We then tested all animals for their innovation propensity in two tests within one week. At this age, the animals are close to sexual maturation. Four weeks after the first behavioural and physiological test round, we repeated all personality and physiological measurements to assess temporal consistency.

Each individual animal did not participate in more than one test per day and was given at least 24 h recovery period after every test. The innovation tests were always presented in the same order (knocking over a cylinder, followed by a stick removal test) because pilot studies had shown that the Cylinder test was easier than the Stick removal test. All other tests were conducted in random order. The Novel object, Open Field, Struggle and heart rate tests were always conducted between 9–11:30 am, blood samples for cortisol were taken at 12 pm (+/− 10 min) following established protocols for cavy-species (Hennessy et al. 2006; Siegeler et al. 2011). Resting metabolic rate measurements lasted 3.5 h and ran between 9 am and 6 pm.

### Innovative problem-solving tasks

First, the easier task (Guenther and Brust 2017), asking the animals to knock-over a transparent cylinder (10 cm high, 3 cm diameter) that was placed on the rough stone inside their home enclosure was performed to ensure that the animals did not lose motivation to participate. The cylinder blocked access to a piece of cucumber, a highly motivating food reward for cavies. Small holes drilled into the cylinder ensured that the animals could smell the food reward. During the one-hour test, the partner animal was removed from the enclosure so that we could observe individual reactions. The experimenter left the room and the test was video recorded. We analysed the latency to first touch of the test setup after the experimenter had left as well as the time to solve (in seconds), whether the task was solved and the number of touches with the test setup – either until solving it or until the end of the observation period (1 h).

The second test followed the same protocol but the actual task was to remove a metal stick from a transparent food tray, causing pieces of cucumber to drop from a platform and become accessible for the animal. The food tray resembled the normal food trays animals knew but was completely transparent. The setup was slightly larger than the animals themselves (20 cm high, 10 × 10 cm ground plate). Both tasks were presented to the animals only on one occasion.

### Personality measures

#### Open field test

The 1 m^2^ sized open field arena was located in another than the housing room of the animals. The focal animal was gently removed from its home enclosure and transported to the arena and introduced beneath a semi-transparent shelter in the middle of the arena. This shelter was left in place for 10 min before being removed remotely. In total, a camera located above the enclosure recorded the distance covered by the animal for 20 min. The videos were analysed using “Labimals (Analysis of laboratory animals, Bielefeld, Germany)”. The setup was cleaned with 70% ethanol after every run. The open field test is generally a stressful experience for rodents and measures the reaction (from passive to active) towards unknown areas, i.e., it is a forced exploration test (Hall and Ballachey 1932).

### Novel object test

To measure response to an unknown object, we used a novel object test. In this species, the response to an unknown object is correlated with aggressiveness (Guenther and Trillmich 2023) and with spatial exploration (Guenther et al. 2014). The novel object test was carried out in the animal’s home enclosure after gently removing the partner animal to measure an individual’s response towards unknown objects. An unknown object was introduced about 30 cm in front of the animal’s shelter. The size of the objects used was adjusted to the actual size of the animals, ensuring that the objects were smaller than the animals reached in height. Thus, we used a round green egg cup (about 4 cm) for the first round and a yellow rubber duck (about 10 cm) for the second round. The animals’ voluntary interactions with the object were video-recorded for one hour. We measured the number of touches within 15 min following the initial touch of the object by the test animal. The number of touches is highly correlated with additional measurements such as the latency to first touch, which we did not analyse further for this reason (Guenther and Trillmich 2013).

### Struggle docility

The struggle test consisted of taking the animal out of their home cage and holding it on its back in one hand. Subsequently, the time that the animal tried to actively escape the hand was measured for 30 s by using a stopwatch. This measurement reflects handling docility and is weakly positively correlated with forced exploration in the open field, i.e., it reflects how an individual copes with a stressful situation (Guenther and Trillmich 2015).

### Physiological measures

### Heart rate

Heart rate was measured using a stethoscope. The animal was picked out of the cage and held until it became calm. Then the heart rate was measured by counting the number of beats during 15 s.

### Resting metabolic rate

The resting metabolic rate was measured using an open-flow respirometer in which two animals could be measured simultaneously. Measurements lasted in total 3.5 h and were conducted under dim light to calm the animals. The temperature was set to 20 ± 2 °C. Metabolic chambers (18 × 28.5 × 18 cm in size) were covered with wood chips to absorb excretions. Outside air was pumped through cuvettes into two successive coolers for drying (M & C Cooler, Ratingen, Germany) with an air flow of 85 L/h (mass flow meter FM-360, Tylan Corp., Torrance, CA). Thereafter, the air was dried further by flowing through scrubbers (Drierite, Fluka, Steinheim, Germany) before entering the metabolic chamber. As controls, we compared dried outside air against CO_2_ and O_2_ concentrations measured in the outflow of the metabolic chambers (CO_2_ analyzer: Maihak AG, Hamburg, Germany; O_2_ analyzer: Oxzilla FC, Sable Systems, Henderson, NV). The analysis software was set to an initial control of 10 min to allow the system to stabilise. Thereafter, O_2_ consumption and CO_2_ production were measured for eight periods of 10 min each, interspersed with 1-min control intervals to allow correction for eventual drifting of the system. The RMR (KJ/day/kg) was calculated from the 3-min interval with the lowest, stable oxygen consumption throughout measurement periods 3–8. Before the metabolic measurements, each individual was weighed to allow the calculation of mass-specific metabolic rate.

### Statistical analyses

For statistical analysis and graphs, R 4.3.0 (R Development Core Team 2023) within RStudio (version 2023.6.0.421, R Development RStudio Team 2020) was used with the packages lme4 (Bates et al. 2015) and lmerTest (Kuznetsova et al. 2017) for mixed models. To create the graphs, we used the packages ggplot2 (Wickham 2016) and ggsignif (Ahlmann-Eltze and Patil 2021).

To test if the probability to interact (binary variable) with the innovation test set-up depended on size rank (continuous), litter size (continuous) or sex (2 level factor), we used a generalised linear mixed model with binomial distribution and included size rank, litter size, sex and the type of the innovation test (2 level factor) as fixed effects. Mother ID and the ID of the individuals were included as random effects.

For further analyses, we removed non-participating animals since it was unclear whether they would have been able to solve and were just not willing to participate or whether they did not participate because they were not able to solve. We employed three separate generalised linear mixed models with binomial distribution to assess different effects on the solving success (solved yes/no). First, we checked whether the interaction (latency to first touch and number of touches) with the innovation test set-up predicted the solving success. Second, we investigated in two separate models if early-life effects of competition influence the solving success. One model included size rank within the litter and the litter size as fixed effects. Size rank and litter size only show a weak correlation (*R* = 0.29 CI: 0.05–0.49). We therefore decided to include both variables in the model. In the other model, growth rate was fitted as fixed effect. Additionally, all three models included sex and type of innovation task as fixed effects as well as individual ID as random effects. Mother ID was dropped from the model assessing the influence of size rank and litter size because usually only one or maximally two offspring per litter actually solved, thus leading to many singularities for the maternal identity which led to converge issues.

For analyses we log-transformed the data for the resting metabolic rate and cortisol levels and used a square root transformation for the data derived from the open field test to match model assumptions of a Gaussian residual distribution. Data derived from the novel object test resembled a Poisson distribution, thus, we fitted it using the Poisson family argument.

In order to test if solvers and non-solvers differ in physiological or personality traits, we ran 6 separate models each using one physiological (baseline cortisol concentration, resting metabolic rate, heart rate) or behavioural variable (response to an unknown object, response to an unknown environment and docility) as response. Solver or non-solver (solving at least one of the innovation tasks; binary) and test round (as they are repeated measures of two test rounds) were fitted as fixed effects. Individual ID and mother ID were included as random effects.

An overview of all the model results can be found in the supplementary material.

To test for consistent individual differences, we estimated repeatability by using the R-package rptR (Stoffel et al. 2017) with 1000 bootstraps for estimating confidence intervals and a likelihood ratio test for significance testing of repeatabilities. As we wanted to assess individual consistency, we used individual ID as grouping factor in the model. Models for repeatability analyses of physiological and personality traits included test round as fixed effect and were conducted on the entire dataset. However, for testing repeatability of innovation behaviour, we used the subset, containing only individuals that touched the innovation task at least once and included the type of test set-up as fixed effect in a binomial model. For behavioural variables, we expected repeatability estimates of 0.25–0.40 to reflect biologically meaningful individual variation (Bell et al. 2009). While we expected slightly higher repeatability estimates for physiological parameters, too few studies have yet reported repeatability estimates for innovation/problem-solving tasks to make clear predictions regarding the magnitude of the expected repeatability.

Residuals of all models were checked visually for distribution and variance homogeneity using Q–Q plots.

### Cortisol plasma concentration

Blood samples (∼80 µl) were taken within three min after capturing an individual to avoid a rise of baseline concentrations due to handling stress (Romero and Reed 2005). One experimenter held the animal on its lap while a second experimenter collected blood from the marginal ear vein into heparinised capillaries. The blood was immediately centrifuged for 5 min at 8000 rpm and then stored at − 20 °C until further analyses. Analysis was performed in the Department of Behavioural Biology of the University of Münster using a competitive enzyme immunoassay (RE52061 IBL, IBL International GmbH, Hamburg, Germany) using specific antibodies against cortisol (for further details see Kaiser et al. 2003). The antibody that we used cross-reacted with relevant steroids as follows: Prednisolone 29.8%, 11-desoxycortisol 8.48%, cortisone 4.49%, prednisone 2.12%, corticosterone 1.99%, 6b-hydroxycortisol 1.03%. The intra-assay CV was 5% and the inter-assay CV was 7.2%.

## Results

### Innovation behaviour

Overall, most animals actively participated in the innovative problem-solving tasks (participation rates: 77%). The overall solving rate was 44%, with solving rates of 49% for the “knock-over” task and 39% for the stick-removal task. Table 1 gives a detailed overview across both tasks.

**Table 1:** Overview of the number of animals that solved or did not solve the two innovation tasks “Knock-over” and “Stick removal” for young independent individuals. The animals that did not solve the innovation task were split into individuals that interacted with the test set-up and the ones that did not touch the test-setup. Note that four individuals accidentality overthrew the test set-up for the Stick-removal task, rendering it non-solvable. These four animals were removed from the analyses

	Knock-over	Stick-removal
Sample size overall	69	65
Solver	26	16
Non-solver	43	49
Non-solver & no interaction	16	24
Non-solver & interaction	27	25

Neither size rank (β = 0.72, SE = 0.81, z = 0.89, *p* = 0.37) nor litter size (β = 0.002, SE = 0.84, z = 0.002, *p* = 1) or sex (β = − 1.21, SE = 1.22, z = − 0.99, *p* = 0.32) influenced if an individual participated in the task or not.

Considering only the animals that participated in the test, innovation behaviour exhibited no repeatability across the two tests (*R* = 0.19, CI: 0.19, *p* = 0.1, Table 2).

**Table 2:** Overview of repeatabilities (R) with corresponding confidence intervals (CI) and p-values for the investigated physiological as well as behavioural traits. Shown are raw repeatabilites accounting for the test setup respectively testing round and including individual IDs as grouping factor in the model. The repeatability for innovation was calculated including only the animals that participated in the innovation task, i.e. touching the innovation set-up

Trait	Variable	Model distribution	*R*	CI	*p*
Innovation	Solving yes/no	Binomial	0.19	0.19	0.10
Cortisol	Log (baseline plasma cortisol concentration)	Gaussian	0.12	0–0.36	0.15
Heart rate	Number of heart beats within 15 s	Gaussian	0.35	0.11–0.53	0.001
Resting metabolic rate	KJ/day/kg	Gaussian	0.51	0.31–0.66	< 0.001
Struggle docility	Time struggling	Gaussian	0.38	0.14–0.56	< 0.001
Open field	Sqrt (distance covered in open field)	Gaussian	0.52	0.33–0.67	< 0.001
Novel object	Number of touches	Poisson	0	0–0.28	0.65

### Is solving success predicted by interaction, i.e., persistence, with the task?

In the next step, we analysed whether the interaction with the test setup of the innovation tasks influenced the problem-solving probability.

**Fig. 1:**
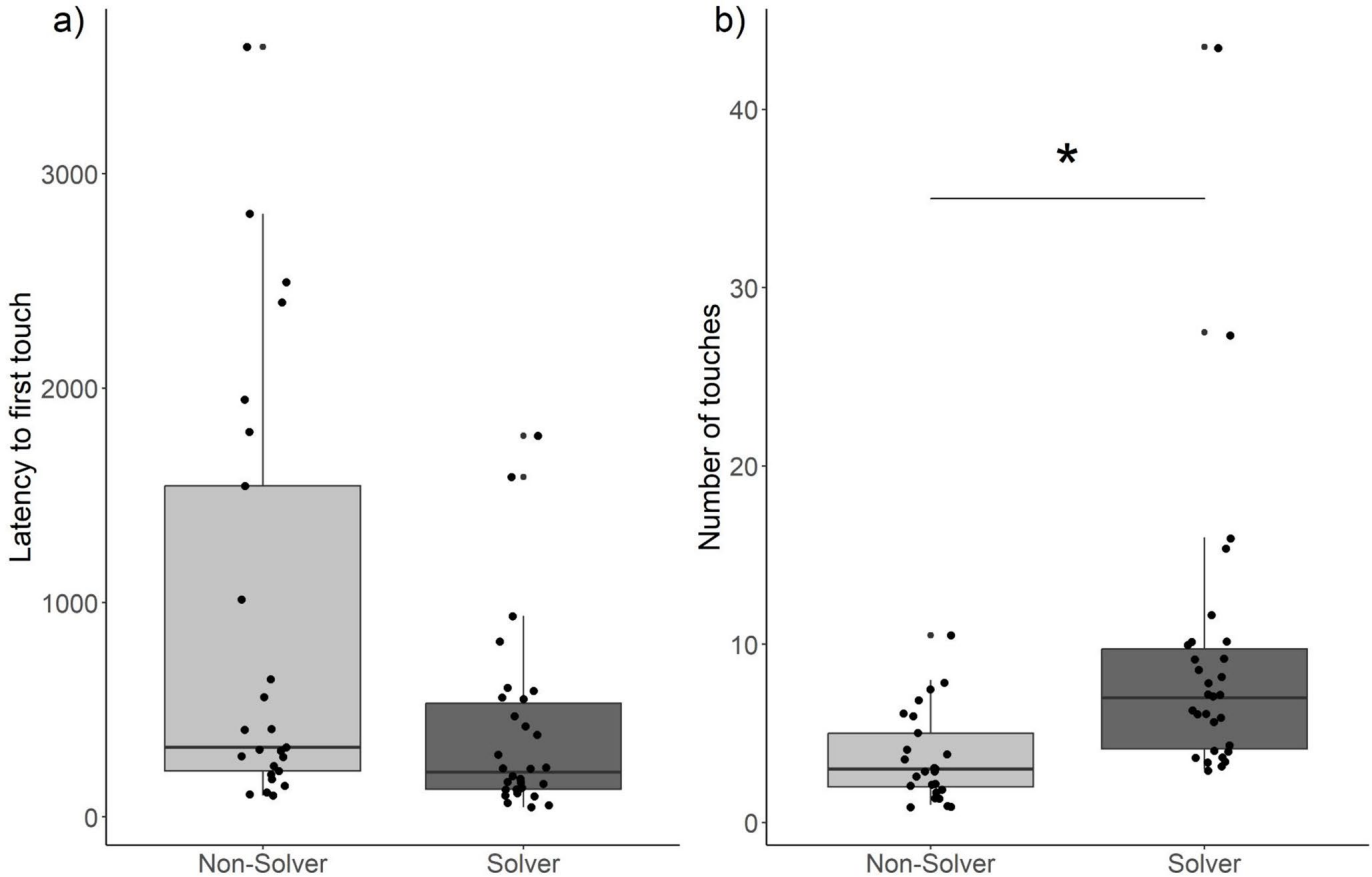
Latency to touch the innovation test set-up (**a**) and the number of touches (**b**) for non-solvers (light grey, *n* = 25) and solvers (dark grey, *n* = 30). Boxes indicate the inter quartile range (IQR), with the central line depicting the median and the whiskers extending to 1.5*IQR. The dots represent the means of the raw data for each individual. The asterisks indicate significant differences

There was a trend that the latency to touch the innovation task negatively affected the success rate to solve the task. In other words, animals that took longer to start interacting with the task tended to show a lower probability of solving it. (β = − 1.96, SE = 1.01, z = − 1.94, *p* = 0.05, Fig. 1). Likewise, the more interactions an individual had with the task, the higher the probability to solve (β = 1.45, SE = 0.59, z = 2.5, *p* = 0.01, Fig. 1).

### Is solving success predicted by early-life effects of competition?

We tested if early-life features like litter size, size rank and growth rate had an influence on the propensity to solve an innovation task.

Neither litter size (β = − 0.22, SE = 0.43, z = − 0.52, *p* = 0.6) nor the size rank (β = 0.44, SE = 0.4, z = 1.11, *p* = 0.27) or growth rate (β = 0.09, SE = 0.27, z = 0.34, *p* = 0.74) significantly influenced the propensity to solve an innovation task suggesting no influence of early sibling competition on the likelihood of becoming a solver.

### Do solvers and non-solvers differ in physiological traits?

Heart rate as well as resting metabolic rate showed significant repeatability and thus consistent individual differences, while cortisol did not (Table 2).

Animals that solved at least one innovation task did not differ in any of the physiological traits from the animals that did not solve any of the tasks (cortisol: β = − 0.19, SE = 0.14, t = − 1.33, *p* = 0.19; heart rate: β = 5.09, SE = 4.65, t = 1.09, df = 90, *p* = 0.28; resting metabolic rate: β = 0.02, SE = 0.08, t = 0.22, df = 50.2, *p* = 0.83).

### Do solvers and non-solvers differ in behavioural traits?

The time an animal spent struggling as well as the distance an animal moved in an open field showed significant repeatability, indicating that they represent personality traits (Table 2). The number of contacts with a novel object on the other hand showed no significant repeatability (*R* = 0, CI: 0–0.28, *p* = 0.65, Table 2).

**Fig. 2:**
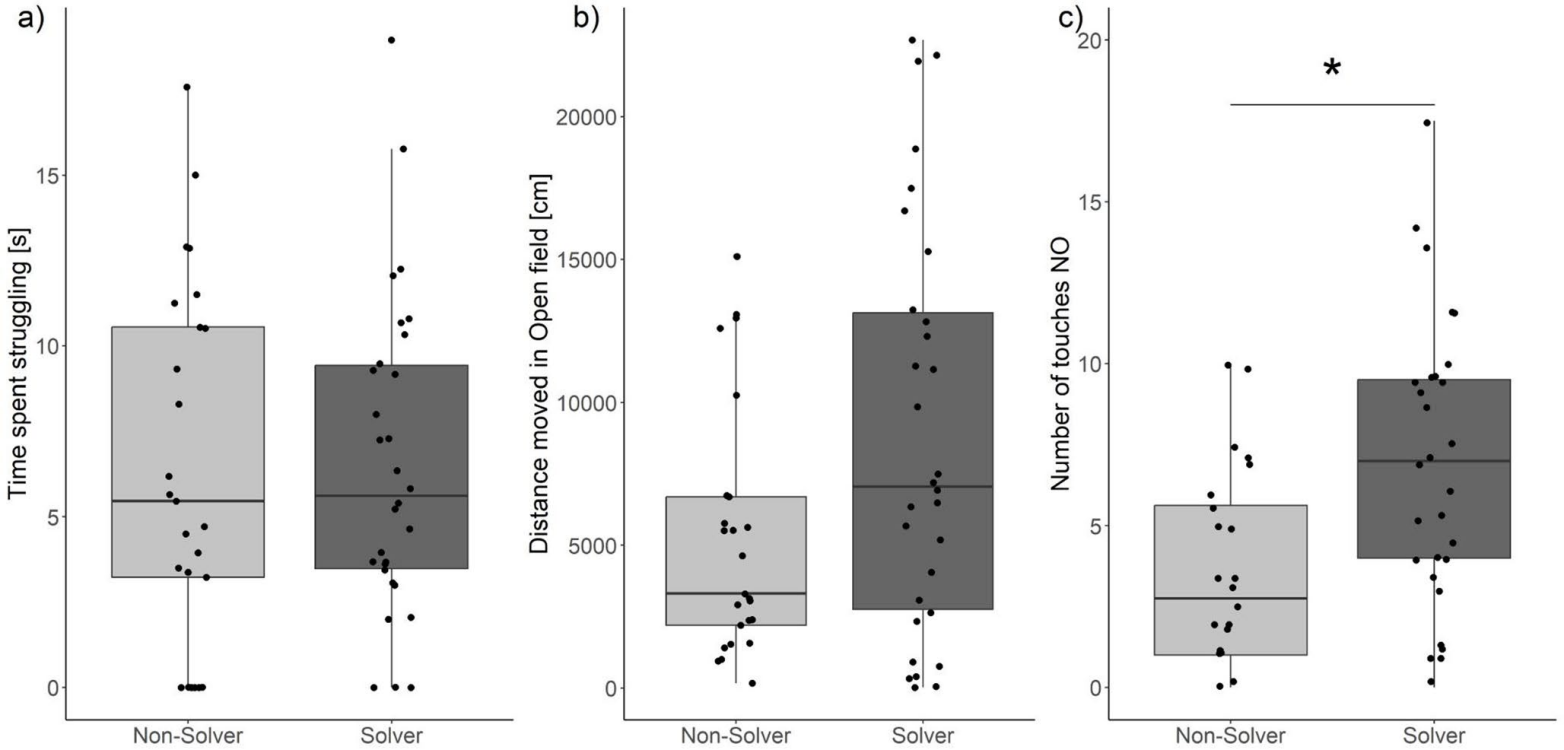
Struggling docility (**a**), distance moved in an open field (**b**) and interaction with a novel object (**c**) for non-solvers (light grey, *n* = 25) and solvers (dark grey, *n* = 30). Boxes indicate the inter quartile range (IQR), with the central line depicting the median and the whiskers extending to 1.5*IQR. The dots represent the means of the raw data for each individual. The asterisks indicate significant differences

While solvers and non-solvers did not differ in their struggle docility (β = 0.73, SE = 1.26, t = 0.58, df = 50.9, *p* = 0.56) and the distance moved in the open field (β = 10.04, SE = 9.46, t = 1.06, df = 48.4, *p* = 0.29), animals that had many interactions with the novel object in a novel object test were more likely to solve the innovation tasks (β = 0.71, SE = 0.23, z = 3.14, *p* = 0.002, Fig. 2).

## Discussion

We tested the hypotheses that early competition, specifically scramble competition among siblings, would affect which individuals innovate at higher rates later in life. Furthermore, we tested if intrinsic physiological or behavioural characteristics differ between individuals who are innovative versus individuals who are not. We did not find evidence that early life competition influences who becomes an innovator. Since previous studies in this species, using comparable or even lower sample sizes, found effects of e.g. size rank in the litter on behavioural and physiological traits (Guenther and Trillmich 2015; Kraus et al. 2024), the absence of significant effects likely mean that innovate problem solving is not influenced by early-life competition. Physiological traits did not differ between solvers and non-solvers, although we showed that most tested traits (except cortisol) differ consistently between individuals throughout the test period. Likewise, behavioural traits related to how individuals react to stressful situations (struggle docility and Open Field test), did not differ between solvers and non-solvers, despite showing consistent individual differences. Reaction to novelty, although not repeatable, predicted success in innovation. Animals interacting more often with novel objects generally and the innovation tasks in particular, were more likely to solve.

The participation rate in our study was 77% and the solving rate was 49% for the easier of the two tasks in young, recently independent animals. These results fit well into what is known from other rodent species, with participation rates of animals tested in captivity ranging between 75 and 96% and reported solving rates for comparable tasks between 25% up to 78% (Guenther and Brust 2017; Mazza and Guenther 2021; Vrbanec et al. 2021; Rowell and Rymer 2022). A comparison across taxa is difficult at this stage due to usually very different test conditions and because participation rates are not reported in most studies. In birds and mammals, innovation has been shown to increase the likelihood of adjusting to human-altered environments as well as enhance success in colonising and establishing in novel environments (Sol et al. 2002; Ducatez et al. 2013; Kozlovsky et al. 2015).

The wild cavy is a commonly found grassland species distributed across wide parts of South America. It has been reported close to human settlements and is even found in parkland areas within the city of Buenos Aires (Acosta and Pafundi 2005; Cavia et al. 2009), demonstrating their potential to successfully colonise and adjust to human-altered landscapes like all rodent species tested for innovation so far. Thus, it comes as no surprise that participation and solving rates are comparable. Studies on species not successfully colonising human-altered environments are lacking but would sorely be needed to gain a better understanding of this behaviour in contexts other than human disturbance.

Inferior competitive abilities are discussed to drive innovation behaviour (Laland and Reader 1999; Amici et al. 2019). According to the ‘bad-competitor hypothesis’, individuals with poor competitive abilities should show enhanced innovation tendencies to exploit uncommon resources. Currently, this has exclusively been tested in adult animals (e.g. Scantlebury et al. 2008; Bandyopadhyay et al. 2026), thus, we lack an understanding of when during life such differences in competition might shape innovation propensity. In polytocous birds and mammals, siblings of the same reproductive event often differ in birth mass or size and heavier/larger young are often able to obtain a greater share of parental resources such as milk (Fey and Trillmich 2008) or a thermoregulatory better position among siblings in the nest (Bautista et al. 2008). Usually, these benefits are realised by sibling competition. Here, we tested the idea that such differences in early-life competitive abilities might shape innovation behaviour. Neither the size rank within a litter, nor litter size (with larger litters representing a more competitive early environment), or growth until weaning, predicted innovation propensity after the onset of independence but before entering the reproductive life stage. Similarly, a study in fawn-footed mosaic-tailed rats reported no effect of maternal care on later offspring innovation (Rowell and Rymer 2022). Although the evidence is still (with this being only the second study testing effects of the early family environment) very anecdotal, these studies might suggest that competition during the period of dependency on parental behaviour does not drive innovation propensity.

Knowing that innovation propensity is often repeatable in adult individuals, i.e. individuals differ consistently from each other (rodents: Mazza and Guenther 2021; Rowell and Rymer 2021; Vrbanec et al. 2021; birds: Morand-Ferron et al. 2011; Miller et al. 2022) and potentially even heritable (rodents: Rowell et al. 2021; birds: Quinn et al. 2016), the question remains when during life and by which factors innovation behaviour is shaped. A recent meta-analysis suggested that life stage/age may influence repeatability (Cauchoix et al. 2018), often with younger animals showing lower repeatability because several cognitive functions are not yet fully developed. This might explain why we did not find repeatability to differ from zero here, since we tested young, not fully adult animals. Rowell et al. (2021) argue in their review article that innovation or problem-solving is likely shaped by an interaction of (epi-) genetic and neuroendocrine factors throughout development. Extrinsic factors, including both, the abiotic and the social environment, are hypothesised to influence the ontogenetic development of innovation. Several effects such as food availability/quality, predation pressure etc. are well-known to affect development after the onset of independence. Innovating might help exploit limited resources in harsh environments and may thus be selected for. Mountain chickadees *Poecile gambeli*, for example, living in harsher high elevation montane habitats with longer winters solved novel foraging problems significantly faster than chickadees living at lower elevations, most likely because finding food in these habitats was more challenging, and survival depends on plastic responses to these challenges (Kozlovsky et al. 2015). Despite larger brain volumes, house mice (*Mus musculus domesticus*) from high-quality nutritional environments performed worse in a series of three innovation tasks than mice fed on a lower quality food, suggesting that harsh environments might boost innovation (Gorshkova et al. 2024). Alternatively, or in addition to such early life effects, competition during adult life may shape innovation capacity.

Competition among adults is often for reproductive opportunities. Males of many species follow several alternative reproductive tactics to gain access to females (Taborsky 2008). In such species, larger, more competitive males often defend territories while smaller and less competitive males employ roaming or sneaking tactics to avoid aggressive interactions (Schradin and Yuen 2011). In African striped mice, older dominant males were more motivated in an escape paradigm to return to their nests and join their female partners and offspring (Rochais et al. 2021). In house mice, roamer males innovate at higher rates than territory holders (Bandyopadhyay et al. 2026) in voluntary food-extractions tasks. Interestingly, when tested outside of a potentially active reproductive context and housed singly in cages, differences in innovation propensity are lost in male house mice. This might suggest that innovation is strongly shaped by challenges throughout adulthood.

Previous studies in birds reported differences of glucocorticoid concentrations (specifically corticosterone) between solvers and non-solvers, albeit in complex ways (Bókony et al. 2014). House sparrows with higher feather corticosterone levels, presumably those, that experienced more stress during the previous moult period and/or were more stress sensitive, performed worse in cognitively challenging problem-solving tasks. Those tasks that showed a negative effect of elevated corticosterone, required enhanced inhibitory control. Likewise, learning efficiency, measured as a decrease in solving time, were better in birds with lower corticosterone levels (Bókony et al. 2014). The authors interpreted these results as being in line with general findings that high corticosterone levels suppress working memory and filter out irrelevant information (McEwen and Sapolsky 1995). On the contrary, a study in greylag geese (*Anser anser*) found a positive effect of elevated corticosterone in faecal samples, which the authors interpreted as mild stress enhancing foraging innovativeness (Pfeffer et al. 2003). Only one study investigated the relationship between corticosterone (glucocorticoid metabolites from faecal samples) with innovative problem-solving (Rowell et al. 2022). Comparable to our own results, they also found no relationship of corticosterone with the number of problems solved or the latency to solve, concluding that problem-solving mainly relies on other hormonal drivers. Taken together, these few but contradictory results might suggest that hormonal (specifically glucocorticoid) influences on innovation are species- or taxon- specific or that effects only become visible in stressful environments and/or test situations but that innovative problem-solving per se is not directly related to the activity of the HPA-axis. In addition to glucocorticoid concentrations, we investigated further physiological parameters, namely heart rate and resting metabolic rate. Both traits showed consistent individual differences but were not related to innovation. The necessity drives innovation hypothesis suggests that physiological condition related to energy reserves may be directly linked to innovation because it influences competitive ability (Laland and Reader 1999). Accordingly, animals in poor condition, i.e., with low energy reserves, should be better innovators. This was, however, not supported using indirect measures of energy reserves (Boogert et al. 2008; Overington et al. 2011; Thornton and Samson 2012). Using direct physiological measures of metabolism, Bókony et al. (2014) showed that house sparrows with higher oxidant concentrations were better at solving some, albeit not all innovative problem-solving tasks. Glucose is the most important energy source for the brain and hence cognitive processes (McNay et al. 2000). In African striped field mice (*Rhambdomys pumillo*), higher blood glucose levels reduced attention, spatial memory and learning (Rochais et al. 2019) but showed no relationship with innovation propensity (Rochais et al. 2021). These results, albeit very few, may suggest that physiological aspects related to energy reserves do not directly drive innovation. Since all studies tested different species as well as different physiological parameters, more studies will be needed before a final conclusion can be drawn.

Innovation propensity has sometimes been described to be linked to personality differences. The general pattern indicates that innovation is more common in more explorative and/or neophilic animals (Amici et al. 2019). Exploratory individuals often have higher interaction rates with problems, increasing their likelihood of solving innovative tasks. Explorative fawn-footed mosaic-tailed rats (*Melomys cervinipes*) were faster problem-solvers than their shy conspecifics (Rowell and Rymer 2021) while neither exploration nor boldness directly predicts problem-solving in house mice (Vezyrakis et al. 2025). Moreover, individual differences in how likely an animal is to approach a problem-solving task (i.e. a novel object) can affect the ability of an individual to participate in a problem-solving task. Adult male Carib grackles (*Quiscalus lugubris*) that solved a problem-solving task were less neophobic and more exploratory than non-solving individuals (Overington et al. 2011). Here, we could show that in line with most studies, individuals that interacted with novel objects generally and the innovation tasks in particular, were fast and more likely to solve the task.

In conclusion, we show that behavioural innovativeness in a precocial rodent is unlikely to be constrained by early development but largely depends on how individuals react to novelty.

## Supplementary Information

Below is the link to the electronic supplementary material.

Supplementary Material 1

## Data Availability

The raw data associated with this article have been uploaded to the data server of Bielefeld University and will be publicly available under 10.4119/unibi/3019205.
